# Our New York Letter

**Published:** 1886-06

**Authors:** W. F. Westmoreland

**Affiliations:** New York


### Correspondence.

### OUR NEW YORK LETTER.

*Editors Atlanta Medical and Surgical Journal:*

I don't think I could write anything more interesting than a visit to the surgical wards of Roosevelt Hospital. While there I saw Dr. Sands, surgeon to the hospital, perform several operations, one of which, a removal of a floating cartilage from the knee joint, was of special interest.

Dr. Sands' method of operation is different from the usual mode. He finds the cartilage and transfixes it with an awl (a fine surgeon's needle mounted in a handle). This he considers very important, as it prevents the cartilage from becoming lost, and he never operates without accomplishing this. The opening into the joint was made in a different line from the external incision, so that the latter was not over, but to one side of the former. The cartilage was removed from the joint with a tenaculum. The spray was not used, but the incisions were made and wound closed under irrigation with solution of bichloride of mercury (1 to 1,000). No drain was left, and the deeper tissues were not stitched together. The external wound was closed with a continuous cat-gut suture.

The wound was dressed as follows: first two layers of sublimated gauze; over this was placed a pad of wood-wool, which extended around the leg; each of these was bandaged separately with a bandage of sublimated gauze. The leg was then extended on a posterior wooden splint, which was furnished with a foot-piece and extended to the upper third of the thigh; the splint was padded with wood-wool, and the leg securely bandaged to it. The doctor thinks this heavy dressing keeps them more quiet than they would be kept without it. The patient is confined to bed two weeks, at the end of which time the dressing is removed and the patient goes out.

The service is very fine, there being at present eighty-five patients in the surgical wards. Of the many interesting cases I saw, the following may be worthy of note: three excisions of the knee, two of the elbow, one of the head of the femur, one of the wrist, an excision of the whole scapula for an osteo-enchodroma, two cases of gun-shot wounds of the abdomen, an amputation of the breast, and an excision of the tongue by Koch's method—a very interesting array of cases to see one after the other.

The strictest antiseptic precautions are observed; bichloride of mercury is the favorite antiseptic; there have been no bad results from it in the hospital yet. They are very much pleased with wood-wool, and use it made up with bags of sublimated gauze into flat pads of various sizes and about an inch thick. It is the best absorbent they have used, and they prefer it to peat, moss or cotton.

I arrived in Philadelphia in time to attend the commencement exercises of the Jefferson Medical College; this was their 61st annual commencement, and took place at the Academy of Music. There was a very large audience. The display of flowers on the stage was beautiful. There were two hundred and twenty-three graduates. The valedictory address was delivered by Professor Theophilus Parvin.

I saw Dr. P. J. Mays, of Philadelphia Polyclinic, treat several cases of neuralgia by hypodermic injections of thein, the new analgesic; the quick and decided cures were wonderful.

W. F. WESTMORELAND, JR.

New York.

